# Dendrimer size effects on the selective brain tumor targeting in orthotopic tumor models upon systemic administration

**DOI:** 10.1002/btm2.10160

**Published:** 2020-04-14

**Authors:** Kevin Liaw, Fan Zhang, Antonella Mangraviti, Sujatha Kannan, Betty Tyler, Rangaramanujam M. Kannan

**Affiliations:** ^1^ Center for Nanomedicine Wilmer Eye Institute, Johns Hopkins School of Medicine Baltimore Maryland USA; ^2^ Department of Chemical and Biomolecular Engineering Johns Hopkins University Baltimore Maryland USA; ^3^ Department of Materials Science and Engineering Johns Hopkins University Baltimore Maryland USA; ^4^ Department of Neurosurgery Johns Hopkins School of Medicine Baltimore Maryland USA; ^5^ Department of Anesthesiology and Critical Care Medicine Johns Hopkins School of Medicine Baltimore Maryland USA

**Keywords:** brain tumor targeting, dendrimer, glioblastoma, immunotherapies, tumor‐associated macrophages

## Abstract

Malignant gliomas are the most common and aggressive form of primary brain tumors, with a median survival of 15–20 months for patients receiving maximal interventions. Advances in nanomedicine have provided tumor‐specific delivery of chemotherapeutics to potentially overcome their off‐target toxicities. Recent advances in dendrimer‐based nanomedicines have established that hydroxyl‐terminated poly(amidoamine) dendrimers can intrinsically target neuroinflammation and brain tumors from systemic administration without the need for targeting moieties. The size of nanocarriers is a critical parameter that determines their tumor‐targeting efficiency, intratumor distribution, and clearance mechanism. In this study, we explore the dendrimer size effects on brain tumor targeting capability in two clinically relevant orthotopic brain tumor models, the 9L rat and GL261 mouse models, which capture differing aspects of gliomas. We show that increasing dendrimers from Generation 4 to Generation 6 significantly enhances their tumor accumulation (~10‐fold greater at 24 hr), tumor specificity (~2–3 fold higher), and tumor retention. The superior tumor targeting effect of G6 dendrimers is associated with its reduced renal clearance rate, resulting in longer circulation time compared to G4 dendrimers. Additionally, the increase in dendrimer generation does not compromise its homogeneous tumor distribution and intrinsic targeting of tumor‐associated macrophages. These results validate the potential for these dendrimers as an effective, clinically translatable platform for effectively targeting tumor‐associated macrophages in malignant gliomas.

## 1 INTRODUCTION

Malignant gliomas are the most common and aggressive form of primary brain tumors, accounting for 70% of brain cancer patients.^1^ The annual incidence is 5.26 cases per 100,000 people, with more than 14,000 new cases diagnosed in the United States each year.^2^, ^3^ They are among the most severe types of cancer due not only to their high rates of recurrence and mortality, but also due to their significant impacts on the quality of life and cognitive function of patients.^1^ Despite recent advances in cancer care, treatment options for patients with malignant gliomas remain limited. Current standard of care for newly diagnosed patients includes maximal safe surgical resection followed by intensive radiotherapy and concomitant chemotherapy, leading to median survival times of 15–20 months for patients with glioblastoma. These survival times have improved minimally in the past few decades.^4^, ^5^, ^6^ Innovative new strategies are necessary to address the plethora of challenges facing glioma treatment to deliver more effective therapies.

The field of nanomedicine has provided advances in tumor‐specific systemic delivery of chemotherapeutics to overcome off‐target toxicities of these therapies via formulation into nanoparticles. As opposed to local delivery, which is highly invasive and requires either surgical intervention or catheterization, systemic delivery utilizes blood circulation and the leakiness of the tumor blood vessels to traffic therapies to the target site noninvasively.^7^ However, the efficacy of such nanomedicine‐mediated systemic therapies have generally not translated into clinical successes due to their failure to address critical delivery challenges, including lack of transport across biological barriers, limiting of systemic distribution, and inadequate accumulation in the brain tumor.^8^, ^9^ Therefore, the size and surface attributes of systemic nanoparticles must be carefully engineered to overcome these challenges. To achieve efficient delivery and high accumulation in brain tumors, nanoparticles must achieve long systemic circulation time without significant residence in peripheral organs, while managing to cross the highly heterogeneous blood–brain tumor barrier (BBTB). Previous studies have found that nanoparticles must be less than 20 nm in size to cross the BBTB and less than 7 nm to diffuse freely within the tumor microenvironment.^10^ However, nanoparticles with smaller sizes often have shorter systemic circulation time compared to larger ones due to the size dependence of renal clearance.^11^ Therefore, careful balancing of these factors is critical for achieving effective brain tumor accumulation.

In addition to effective tumor penetration and accumulation, therapies must access the cells of interest to have an effect on the tumor.^12^ Tumor‐associated macrophages (TAMs) have emerged as promising therapeutic targets for cancer treatment due to their abundance within tumors and their critical roles in manipulating the immune environment toward a pro‐tumor state.^13^ Tumors actively recruit host macrophages and monocytes and repolarize them into TAMs, which suppress immune activation and promote tumor growth, metastasis, and drug resistance.^14^, ^15^, ^16^ Therefore, immunotherapies that can reprogram TAMs from a pro‐tumor to an anti‐tumor phenotype can inhibit their tumor‐supporting functions while simultaneously bolstering their immune activation and antigen‐presenting functions.^17^, ^18^ Based on strong preclinical results, TAMs‐focused interventions are undergoing clinical trials alone or in conjunction with traditional treatment modalities (NCT02829723, NCT01349036, NCT02584647, and NCT03708224). However, translation of immunotherapies targeting macrophages has been limited by low response rates, drug resistance, and systemic toxicities associated with nonspecific immune modulation.^19^, ^20^, ^21^ Therefore, delivery strategies that can carry immunotherapy payloads into the tumor and specifically to TAMs while remaining inactive in the rest of the body may yield positive clinical outcomes.

Poly(amidoamine) (PAMAM) dendrimers are highly tailorable, branched macromolecules in the sub‐10 nm size scale that have been explored as targeting vectors for cancer‐specific treatments and diagnostics.^22^, ^23^, ^24^ We have previously shown that hydroxyl‐terminated PAMAM dendrimers are able to cross impaired blood–brain barriers and selectively target activated microglia from systemic circulation in a variety of models for neurodegenerative diseases.^25^, ^26^, ^27^, ^28^, ^29^ This hydroxyl dendrimer platform has significant potential for clinical translation due to its well‐tolerated nature in vivo^30^ and scalability^31^ and is currently undergoing an early‐stage clinical trial for a pediatric central nervous system indication (NCT03500627). We have previously reported in the 9L rat model of gliosarcoma that systemically administered hydroxyl‐terminated Generation 4 dendrimers (G4) with ~4.3 nm size and neutral surface charge cross the BBTB to penetrate uniformly throughout the solid brain tumor and specifically target TAMs.^22^ However, G4 dendrimers are also quickly cleared from circulation within 24 hr through renal clearance. In this study, we explored whether Generation 6 hydroxyl‐terminated dendrimers (G6) of similar surface charge but greater size (~6.7 nm) can achieve greater tumor accumulation while maintaining the favorable in vivo transport properties of G4 dendrimers. We investigated this in two clinically relevant brain tumor models that capture differing aspects of gliomas: the 9L rat and GL261 mouse models.

## 2 RESULTS

### 2.1 Increasing dendrimer generation enhances tumor accumulation in the 9L model of gliosarcoma

To investigate how dendrimer generation and, by proxy, size, affects their transport kinetics and magnitude of their tumor targeting, we intravenously injected fluorescently labeled G4 and G6 dendrimers separately into different rats bearing 9L gliosarcoma brain tumors (Figure S1). G4 dendrimers are 4.3 ± 0.2 nm in diameter, while G6 dendrimers have ~4‐fold greater molecular weight and a size of 6.7 ± 0.3 nm (Table 1). Both G4 and G6 dendrimers exhibit near neutral surface charge (G4 ζ‐potential = 4.5 ± 0.1 mV; G6 ζ‐potential = 0.25 ± 0.4 mV).

**TABLE 1:** Molecular weight, size, and ζ‐potential of G4‐OH and G6‐OH

Dendrimer	Mw (Da)	Hydrodynamic diameter (nm)	ζ‐potential (mV)
G4‐OH	~14,000	4.3 ± 0.2 nm	4.5 ± 0.1 mV
G6‐OH	~58,048	6.7 ± 0.3 nm	0.25 ± 0.4 mV

**FIGURE 1:**
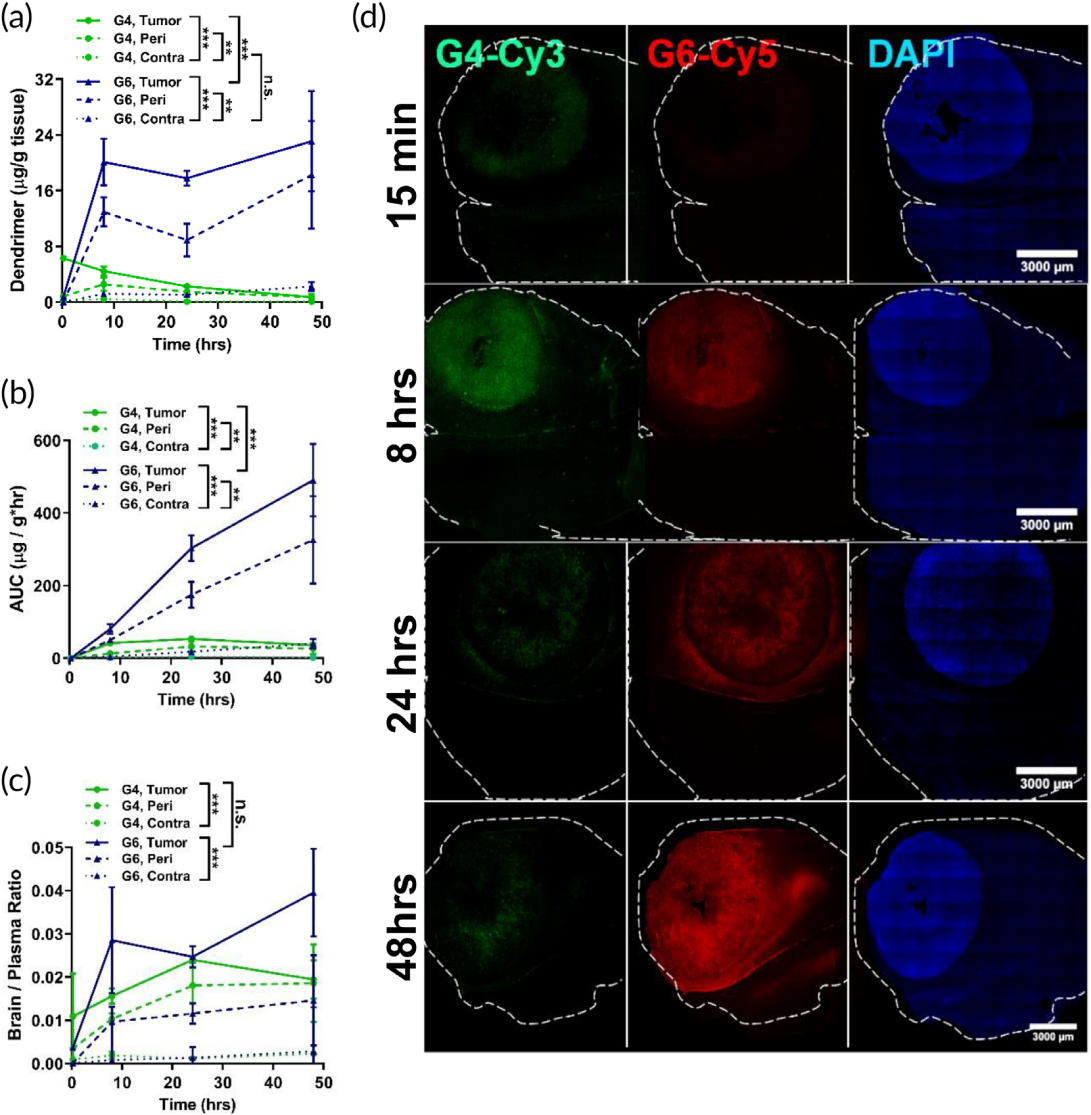
Comparison of G4 and G6 kinetics in gliosarcoma. Fluorescently labeled G4 and G6 dendrimers were injected intravenously into 9L gliosarcoma brain tumor‐bearing mice. (a) G6 dendrimers (blue, solid triangle) exhibit increased accumulation (15 min, 8, 24, 48 hr) in the tumor (solid line) and peritumor areas (dash line) compared to G4 dendrimers (green, solid circle) while maintaining low levels in the contralateral hemisphere (dotted line). (b) Area under the curve (AUC) calculations for G4 and G6 dendrimers based on Figure 1a. (c) G6 dendrimers improve tumor/plasma ratio compared to G4 dendrimers. (d) Confocal imaging of G4 (green) and G6 (red) dendrimer distribution in the tumor‐bearing brain. The tumor mass is indicated by nuclear DAPI staining (blue). Scale bar = 3 mm. *** *p* < .001, ** *p* < .01, ns *p* > .1

To study tumor accumulation kinetics in gliosarcoma, 9L‐tumor bearing rats were systemically administered with dendrimers at 27.5 mg/kg, and tissues were collected for analysis at 15 min, 8, 24, and 48 hr postinjection. G6 exhibited significantly higher tumor accumulation compared to G4, with ~30‐fold greater tumor uptake at 48 hr postadministration than G4 (Figure 1a). The concentration of G6 within the tumor continued to increase in the initial 8 hr, followed by a plateau where G6 uptake was maintained at above 20 μg/g tissue until 48 hr. In contrast, G4 exhibited faster tumor extravasation, with higher levels (6.30 ± 0.1 μg/g) than G6 (0.77 ± 0.19 μg/g) at 15 min. This fast extravasation was followed by declining tumor levels until 0.75 ± 0.27 μg/g at 48 hr. Both dendrimers exhibited high tumor specificity, with significantly greater concentrations within the tumor and the peritumoral area compared to the contralateral hemisphere. At 48 hr postinjection, G6 exhibited ~7‐ and ~2‐fold greater area under the curve (AUC) and tumor/plasma ratio than G4 (Figure 1b,c), indicating greater tumor exposure and tumor penetration.

We then explored dendrimer distribution within the tumor‐bearing brain by injecting equivalent doses of both G4 and G6 dendrimers simultaneously into the same animals (Figure 1d). Corroborating the quantification data, G4 (shown in green) distributes throughout the brain tumor by 15 min postinjection and showed lower accumulation at 24 and 48 hr compared with 8 hr postinjection. Extravasation of G6 into the tumor was slower than G4, with little G6 signal observed within the tumor 15 minutes after injection. However, G6 signal continued to increase in the tumor throughout the next 48 hr, indicating superior tumor accumulation.

### 2.2 TAMs targeting ability is retained from G4 to G6 in the 9L gliosarcoma model

**FIGURE 2:**
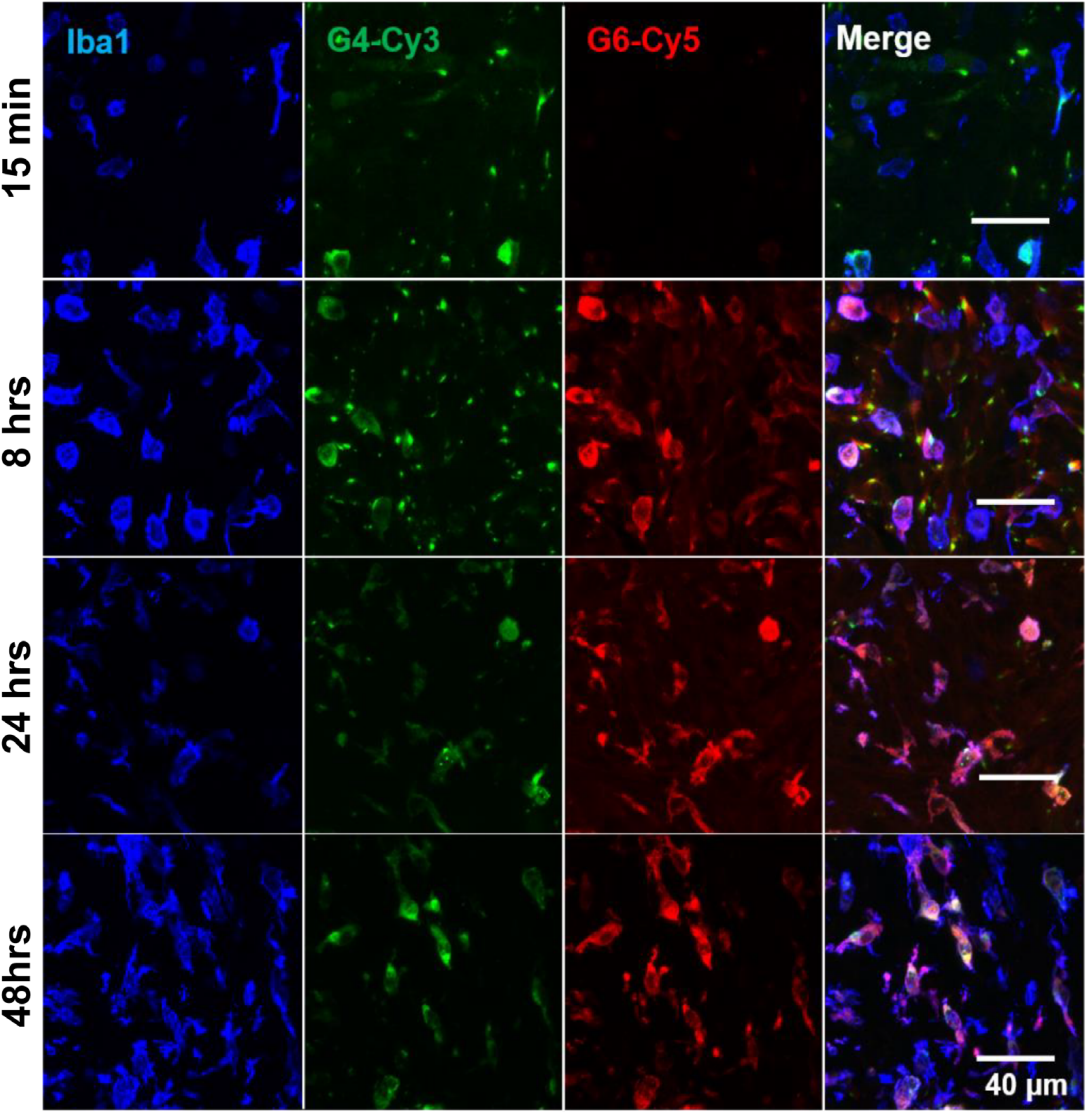
The kinetics of dendrimer uptake in tumor‐associated macrophages (TAMs) is size dependent in gliosarcoma. G4 dendrimers (green) exhibit faster uptake by TAMs than G6 dendrimers (red), with cell‐associated signal within 15 min after injection. G6 dendrimers maintain high accumulation within TAMs (blue) up to 48 hr postinjection. Scale bar = 40 μm

We have previously reported that G4 targets TAMs in the 9L gliosarcoma model. To determine whether this property is retained upon increasing dendrimer generation to G6, we injected G4 and G6 dendrimers into 9L tumor‐bearing rats and stained brain slices with Ionized calcium binding adaptor molecule 1 (Iba1) to label TAMs for confocal imaging (Figure 2). G4 dendrimers exhibited the expected co‐localization with Iba1+ cells, while increasing dendrimer generation from G4 to G6 did not alter this TAMs targeting property. The altered uptake kinetics between G4 and G6 seen in Figure 1 were reflected in the timing and quantity of intracellular dendrimer signal within TAMs. G4 signal was present within TAMs in the tumor as soon as 15 minutes. G6 exhibited slower TAMs uptake, with little signal seen at 15 minutes but strong signal colocalization with TAMs after 8 hr and maintained to the later time points. Both G4 and G6 dendrimers colocalized highly and specifically with Iba1+ cells.

### 2.3 G6 dendrimers show prolonged circulation time and reduced renal clearance in the 9L rat model of gliosarcoma

**FIGURE 3:**
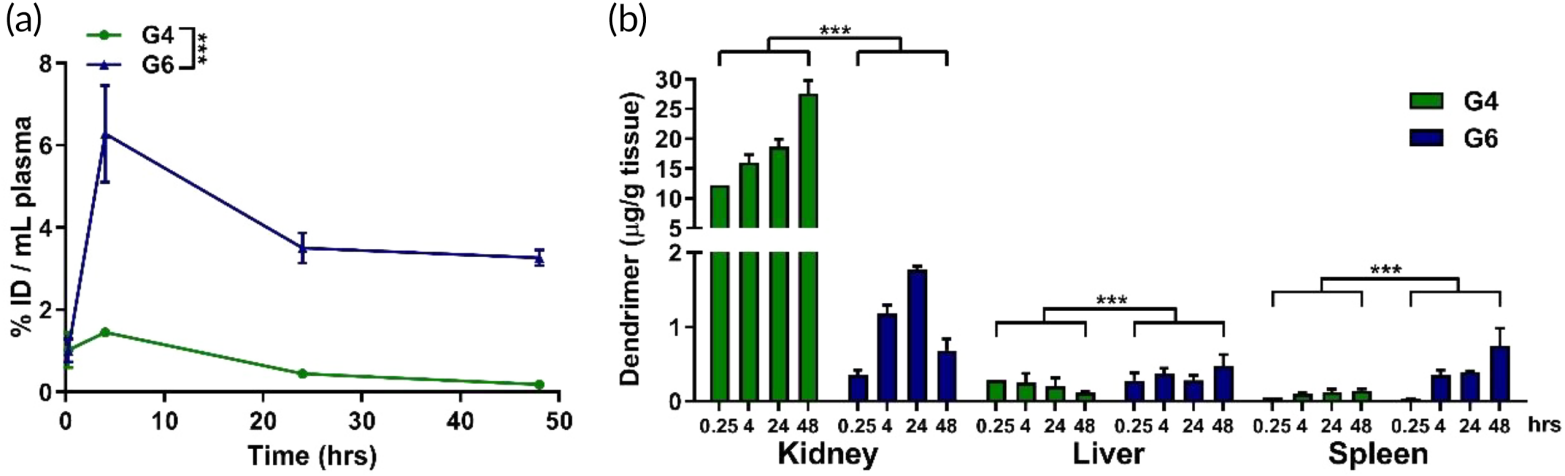
Plasma and systemic biodistribution of G4 and G6 dendrimers. Fluorescently labeled G4 and G6 dendrimers were injected intravenously into 9L gliosarcoma brain tumor bearing mice. Plasma and organs were collected at specified time points, homogenized to extract dendrimers, and measured via fluorescence spectrometry. (a) G6 dendrimers (blue) exhibit higher plasma concentration over time compared to G4 dendrimers (green). (b) Accumulation of G4 and G6 dendrimers in the kidney, liver, and spleen at 0.25, 4, 24, and 48 hr after systemic injection. G6 exhibits significantly lower kidney levels compared to G4, indicating decreased renal clearance. G6 also exhibits slightly increased levels in the liver and spleen due to longer circulation time. ***p < .001

To understand the underlying mechanism for different tumor accumulation between G4 and G6, we compared their plasma clearance and biodistribution in major organs. We found that in plasma, G6 concentration was higher than G4 at all time points (Figure 3a). At 48 hr after injection, G6 exhibits ~18‐fold greater levels (3.26 ± 0.19 %ID/mL) than G4 in the plasma (0.18 ± 0.01 %ID/mL). This increased residence in circulation was associated with reduced renal clearance rate of G6 compared to G4. At 48 hr, there was ~40‐fold lower accumulation of G6 (27.6 ± 2.2 μg/g) than G4 (0.68 ± 0.15 μg/g) in the kidneys (Figure 3(b)). Interestingly, dendrimer accumulation in the liver and spleen remained at low levels for both G4 and G6, indicating renal clearance as the major excretion mechanism for both G4 and G6, albeit G6 had significantly reduced renal clearance rate.

### 2.4 Increasing dendrimer size improves tumor accumulation in the GL261 model of glioblastoma

**FIGURE 4:**
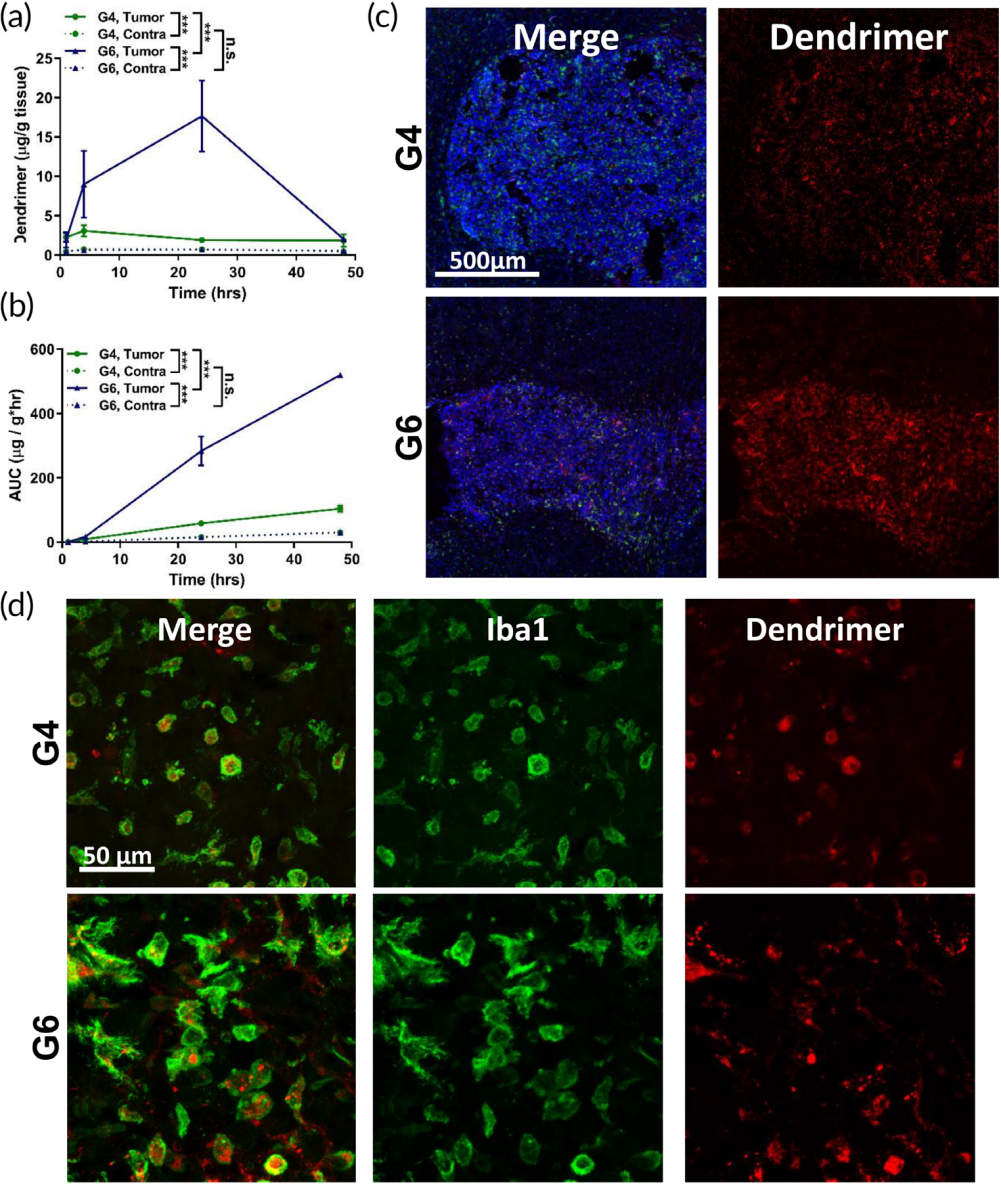
Size dependence of tumor targeting in GL261 murine model of glioblastoma. Fluorescently labeled G4 and G6 were injected intravenously into GL261 brain tumor bearing mice. (a) G6 (blue) dendrimers significantly increases tumor accumulation compared to G4 (green) dendrimers and the contralateral hemisphere. ***p < .001, n.s. *p* > .1. (b) Area under the curve (AUC) calculations based on the data in Figure 4a. (c) G4 and G6 dendrimers (red) penetrate and distribute evenly throughout the solid glioblastoma tumor, with G6 dendrimers exhibiting greater signal within the tumor (indicated by blue DAPI stain) 24 hr after intravenous administration. Scale bar = 500 μm. (d) Both G4 and G6 dendrimers localize specifically within TAMs (green). Scale bar = 50 μm

The 9L rat model of gliosarcoma has been largely used to study the transport of drugs across the blood–brain and blood‐tumor barrier.^32^ However, as a highly immunogenic tumor model, it does not accurately recapitulate the tumor immune prolife of human glioma. We next sought to validate our findings in the immunocompetent GL261 mouse model of glioblastoma, which successfully captures the myeloid cell infiltration and the immuno‐suppressive tumor milieu of human gliomas.^33^ The conversion of dose design between species was based on the surface area difference between rat and mouse models. A final dendrimer dose of 55 mg/kg was used for dendrimer studies in the GL261 mouse model. As in the gliosarcoma model, G6 exhibited significantly greater tumor accumulation than G4 in the glioblastoma tumor (Figure 4a), with ~10‐fold greater tumor levels (G6: 17.6 ± 4.5 μg/g; G4: 1.9 ± 0.3 μg/g) at 24 hr after injection. The tumor accumulation kinetics are slightly different between models. In the GL261 mouse model, G4 concentration in the tumor reached a peak around 4 hr rather than 15 min postadministration as seen in the rat gliosarcoma model. G6 concentration in the tumor continued to increase up to 24 hr and showed significantly greater levels than G4. However, unlike in the gliosarcoma model, we observed a significant decrease in G6 levels after 24 hr, leading to similar tumor concentration with G4 at 48 hr after injection. Further analysis revealed that this decline in tumor accumulation by G6 was attributable to efflux of G6 out of TAMs and into the extracellular space (Figure S2). Image analysis of whole tumor fluorescence signal showed that both G4 and G6 signal within the whole tumor remains constant between 24 and 48 hr (Figure S2a,b). However, further exploration into where the G6 signal in the tumor was located showed that the signal in the extracellular space increased ~3‐fold from 24 to 48 hr after injection (Figure S2c,d). This indicates that G6 dendrimers were cleared from TAMs between 24 and 48 hr but retained within the tumor extracellular space. Our analysis also demonstrated the high specificity of these dendrimers for TAMs, with >80% of dendrimer signal within TAMs at 24 hr. Both dendrimers showed high specificity for the tumor compared to the contralateral hemisphere, with G4 exhibiting ~4‐fold greater levels in tumor compared to contralateral hemisphere and G6 exhibiting ~25‐fold difference at 24 hr. G6 also did not exhibit increased off‐target brain accumulation, with accumulation in the contralateral hemisphere comparable to that of G4. We did not explore the peritumor regions in this model due to size constraints with mice.

AUC calculations based on the quantification data corroborated these trends. G6 exhibited significantly greater tumor AUC (519.8 ± 4.0 μg/g) than G4 (104.1 ± 9.6 μg/g) (Figure 4b). Imaging of G4 and G6 dendrimers in glioblastoma tumor‐bearing mice at 24 hr showed that both dendrimers penetrated throughout the solid tumor and G6 showed stronger fluorescence signal in the tumor (Figure 4c). Both dendrimers exhibit signal specific to the tumor with high fidelity for the tumor border. Additionally, both generation dendrimers exhibited the expected TAMs‐specific localization (Figure 4d).

### 2.5 G6 exhibits increased circulation time and decreased renal clearance in the GL261 model of glioblastoma

**FIGURE 5:**
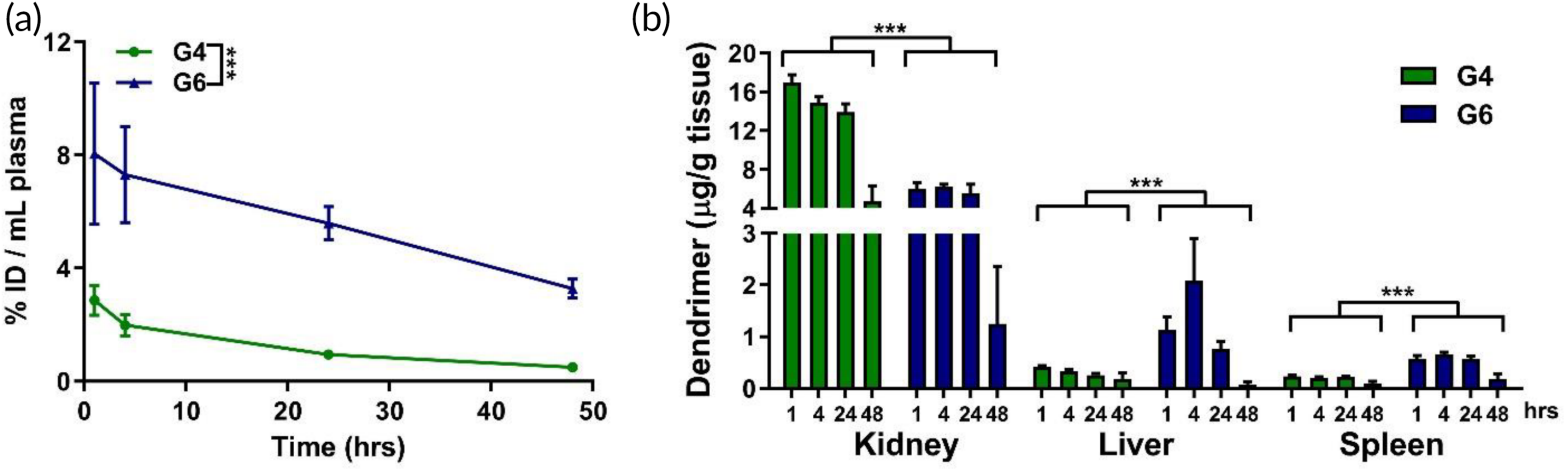
Systemic biodistribution of G4 and G6 dendrimers in glioblastoma. Fluorescently labeled G4 and G6 dendrimers were injected intravenously into GL261 brain tumor bearing mice. Plasma and organs were collected at specified time points, homogenized to extract dendrimers, and measured via fluorescence spectrometry. (a) G6 dendrimers exhibit higher plasma concentration over time than G4 dendrimers. (b) G6 dendrimers have greater levels in the systemic organs compared to G4 due to their longer circulation time except in kidneys, where their increased size enables decreased elimination from renal clearance. ***p < .001

As before, we explored the circulation kinetics and systemic biodistribution of both dendrimers in the glioblastoma model. G6 exhibited prolonged circulation time compared to G4, with ~10‐fold greater levels remaining in plasma after 48 hr (G6: 3.27 ± 0.34 %ID/ml; G4: 0.49 ± 0.04 %ID/ml; Figure 5a). This increased circulation time corresponded to lower kidney levels of G6 compared to G4, indicating reduced renal clearance rate (Figure 5b). Unlike in the gliosarcoma model, we observed increased liver content of G6 compared to G4. However, by 48 hr both dendrimers exhibited less than 0.5 %ID/g in the liver. A similar kinetic profile is seen in the spleen, with both G4 and G6 dendrimers exhibiting less than 0.5 %ID/g at all time points. This indicated that dendrimer‐based delivery vehicles can limit systemic exposure to toxic anti‐cancer therapies compared to freely administered therapies.

## 3 DISCUSSION

In this study, we examined G4 and G6 hydroxyl‐terminated PAMAM dendrimers as vehicles for delivering immunotherapies into brain tumors, selectively to TAMs without the need for any targeting moieties. Both dendrimers accumulated within the tumor and localized in TAMs while exhibiting limited residence in healthy brain tissue and other organs. G6 exhibited significantly greater tumor accumulation in gliosarcoma and glioblastoma models than G4, a property attributed to their longer circulation time resulting from a slowed renal clearance rate. These results demonstrated that using these dendrimers, particularly G6, for targeted systemic delivery of immunotherapies to TAMs may be an ideal strategy to repolarize TAMs for the treatment of brain cancer.

Effective tumor‐targeting from systemic administration requires tumor penetration and long systemic circulation, both of which are highly dependent on nanoparticle size. Dendrimers are an emerging class of polymeric hyperbranched nanoparticles that have been explored recently for targeted brain delivery through both passive and active BBB transport strategies.^34^ Our group has extensively studied G4 PAMAM dendrimers with 4 nm size and neutral surface charge, which effectively target activated microglia without incorporating targeting ligands.^22^, ^25^ In a canine model of hypothermic circulatory arrest‐induced brain injury, we have demonstrated that G6 can improve delivery to regions of neuroinflammation over G4 due to their longer circulation time.^35^ In this study, we hypothesized that G6's hydrodynamic diameter of ~7 nm balances the size‐dependent constraints surrounding effective tumor targeting and renal clearance over G4. To test this hypothesis, we examined G6 dendrimers in comparison to G4 for tumor targeting. Due to physical barriers associated with the BBB and solid tumors, penetration across the BBB and effective diffusion through the dense tumor extracellular matrix have been found to be inversely proportional to nanoparticle size.^8^, ^36^ Bio‐inert nanoparticles in the size range of 50–200 nm have shown limited tumor penetration, while those less than 20 nm in diameter can fully penetrate solid brain tumors.^37^, ^38^ Nanoparticles smaller than 7 nm in diameter have demonstrated unhindered diffusion in brain tumor tissue, although they may also diffuse away and clear quickly from the tumor.^8^, ^10^, ^39^ This indicates that the size dependence of tumor penetration must be carefully balanced with the systemic clearance of nanoparticles.

The size of dendrimers also greatly affects their rate of renal clearance and renal accumulation. Nanoparticles less than 6 nm in diameter undergo rapid renal clearance through the fenestrations in kidney glomerular capillary walls, while those greater than 8 nm are not.^40^, ^41^ Based on these considerations, G6 is able to partially escape renal clearance without altering this as its primary clearance route for prolonged circulation time, which is reflected in the measured plasma and kidney dendrimer content (Figures 3a,b and 5a,b). Many hypotheses propose the glomerular filtration barrier (GFB) as the key barrier to regulate the efficient filtration of macromolecules without clogging.^42^ In one hypothesis, an electrical field is presented across the GFB, preventing charged macromolecules from clogging this barrier.^43^ In another, glomerular based membrane, a dense fibrinous network that lies next to the glomerular endothelial fenestrations, acts as a permeable gel and mediates the filtration of macromolecules through a combination of convective flow and passive diffusion.^44^ In this mechanism, diffusion is regarded as the major anti‐clogging mechanism involved in removing the retentate from the filter. In particular, the theory suggests that smaller size macromolecules partition from the blood into these glomerular membranes to a greater extent compared to larger size macromolecules.^42^ This hypothesis correlates with the higher kidney accumulation of G4 compared to G6 observed in our experiments (Figures 3b and 5b).

Given the significant decrease of renal accumulation resulting from the increase of dendrimer generation, we do not observe any alteration of dendrimer acculturation in the spleen or liver (Figures 3b and 5b). The hepatobiliary system represents the primary route of excretion for particles with sizes beyond the renal filtration cutoff (10–20 nm). Nanoparticles above 40 nm tend to be cleared through the mononuclear phagocyte system, for example, the Kupffer cells.^11^, ^45^ These two mechanisms account for the nanoparticle accumulation in the liver and spleen. Since G6 has a size of ~7 nm, which is below the size range of clearance via the hepatobiliary and mononuclear phagocyte systems, it does not exhibit significant changes in these organs compared to G4.

Taken together, our results demonstrate that G6 exhibits a size optimal for balancing renal clearance and tumor penetration, resulting in greater tumor accumulation in both gliosarcoma and glioblastoma than G4 dendrimers. These findings are consistent with qualitative trends observed in recent studies, which indicate that larger dendrimers and other nanoparticles fail to penetrate the solid tumor while smaller ones rapidly clear out, with the ideal size being in the generation 5–6 range.^7^, ^10^

While dendrimers have been explored for targeting brain tumors, relatively few studies have performed quantitative analysis of dendrimer accumulation in orthotopic brain cancer models.^46^, ^47^, ^48^ Compared to other types of dendrimers injected via tail vein in flank tumor models, our hydroxyl‐terminated PAMAM dendrimer results exhibit similar levels of tumor accumulation despite needing to overcome physical transport barriers in orthotopic brain tumors that flank tumors lack.^49^, ^50^, ^51^ Compared to other types of nanoparticles explored in orthotopic brain tumors, G6 shows a ~100‐fold greater in tumor accumulation than liposomal^52^ nanoparticles and ~10‐fold greater than G4 and reported tumor levels of gold^53^, ^54^ and PEGylated iron oxide^55^ nanoparticles. Studies based on traditional polymeric nanoparticles with ~135 nm size showed only a 50% improvement in the delivery of temozolomide in a rat brain tumor model.^56^ In another study in orthotopic rat brain tumors, liposomes of 80 and 200 nm sizes were unable to penetrate the BBB and distributed heterogeneously in the solid brain tumor, with dense peripheral tumor deposition and incomplete tumor core penetration.^57^ Due to the limitations in passive targeting with these nanoparticles, active targeting strategies are often adopted in larger nanoparticle systems to improve their tumor penetration.^58^ Surface modifications with targeting ligands such as angiopep‐2 and nestin have been employed to achieve even greater tumor accumulation,^59^, ^60^ a strategy we are exploring to further improve the tumor targeting of these PAMAM dendrimers. Additionally, these dendrimers achieve greater specificity for the tumor compared to healthy brain tissue. At 24 hr after injection, G6 dendrimers exhibited ~10‐fold and ~15‐fold greater accumulation in tumor compared to the contralateral hemisphere in the 9L and GL261 models, respectively, while G4 dendrimers exhibited a ~5‐fold greater tumor accumulation in tumor compared to contralateral hemisphere in both models. In comparison, gelatin‐conjugated polylysine dendrimers and PEGylated iron oxide nanoparticles exhibited <3‐fold specificity.^51^, ^55^ In addition to tumor specificity, these PAMAM dendrimers also specifically target TAMs, with >80% of the dendrimer signal in the tumor localized within TAMs (Figure S2a). This means the measured tumor accumulation represents dendrimer quantity not just in the tumor but specifically within the target cells of interest. To our knowledge, no quantitative analyses of nanoparticle accumulation in orthotopic brain tumors have explored that quantification on a cell‐type level. Therefore, the hydroxyl‐terminated PAMAM dendrimers are a nontoxic, translatable targeting platform that not only exhibits high tumor accumulation, but also enables selective delivery to TAMs.

Gliosarcoma and glioblastoma have long been considered clinically indistinguishable and undergo similar treatment regimens.^61^, ^62^, ^63^ However, recent literature suggests that there are a number of critical distinctive characteristics that warrant separate study and, potentially, intervention.^64^, ^65^ The sarcomatous elements in gliosarcoma result in firm, well‐defined tumors while a hallmark of glioblastoma is its diffuse, highly infiltrative tumor border.^65^, ^66^ Despite this invasiveness, glioblastoma is highly localized to the brain while gliosarcoma is metastatic.^65^ Clinically, patients with gliosarcoma face poorer prognoses.^64^ Sarcomatous elements in tumors have been shown to correlate with increased PD‐L1 expression by tumor cells and suppression of the cytotoxic T‐cells.^67^ This suppression creates an anti‐inflammatory immune environment that polarizes macrophages into TAMs, which exhibit efficient phagocytosis and endocytosis.^68^, ^69^, ^70^ This difference in immune environment arising from sarcomatous elements within the tumor may account for the differences between the two models observed with the G6 dendrimer at 48 hr after injection. The increased TAMs activation in gliosarcoma results in greater tumor accumulation and retention due to greater and more efficient internalization activity. In contrast, the G6 levels within TAMs in glioblastoma peak at 24 hr and then drop off as they are exocytosed into the extracellular space. Further exploration of the mechanism behind this difference in TAMs retention is warranted but may arise due to G6 being carried out with the secretion of intercellular signaling extracellular vesicles from TAMs,^71^, ^72^ which may then exhibit strong interactions with the tumor extracellular matrix to result in the signal pattern observed.^73^ The difference in tumor accumulation kinetics in gliosarcoma compared to glioblastoma arising from sarcomatous content may have implications for the design of dendrimer‐mediated interventions and suggests that they should be considered separately for the development of effective treatments.

Anti‐cancer compounds have been shown to induce systemic toxicities, liver toxicity in particular; therefore, the biodistribution and accumulation of nanoparticles must be considered in addition to their effectiveness in targeting the disease site.^74^, ^75^ Renal filtration is the primary route of clearance for G4 and G6 dendrimers, so, as expected, both dendrimers are present at their highest levels in the kidneys. For all other organs, both dendrimers do not exhibit accumulation and by 48 hr show less than 0.5 %ID/g, indicating limited systemic exposure to the therapeutic payload. This is in contrast to free drug administration of chemotherapies, which quickly clear from tumor tissue while accumulating in the liver at much greater concentrations.^60^, ^76^ These PAMAM dendrimers also compare favorably to gold nanoparticles, PEGylated iron oxide nanoparticles, and other classes of dendrimers, which exhibit slightly higher kidney levels but a significantly greater liver accumulation of 2–70% of the initial dose retained.^49^, ^50^, ^54^, ^55^, ^77^ Taken together with their tumor accumulation results, this biodistribution profile indicates that these dendrimers may yield a large therapeutic window for effective and safe treatments for brain cancers.

## 4 MATERIALS AND METHODS

### 4.1 Materials

Hydroxyl‐terminated Generation 4 (G4) and Generation 6 (G6) poly(amidoamine) (PAMAM) dendrimers were purchased from Dendritech (Midland, MI). Cy3‐ and Cy5‐mono‐NHS esters were purchased from GE Healthcare (Chicago, IL). Benzotriazol‐1‐yl‐oxytripyrrolidinophosphonium hexafluorophosphate (PyBOP), *N*,*N*‐diisopropylethylamine (DIEA), dimethylformamide (DMF), Piperidine, dimethyl sulfoxide (DMSO), trimethylamine, 6‐Fmoc‐GABA‐OH, Triton X, and bovine serum albumin were purchased from Sigma‐Aldrich (St. Louis, MO). Fischer 344 rats were purchased from Harlan Bioproducts (Indianapolis, IN), and 9L gliosarcoma cells were purchased from the Brain Tumor Research Center of UC San Francisco (San Francisco, CA). C57BL/6 mice were purchased from The Jackson Laboratory (Bar Harbor, ME), and GL261 murine glioblastoma cells were purchased from the DTP/DCTD/NCI Tumor Repository (Bethesda, MD). RPMI, fetal bovine serum, penstrep antibiotic, l‐glutamine, and normal goat serum (NGS) were purchased from ThermoFisher (Waltham, MA). Tris‐buffered saline (TBS) and phosphate buffered saline (PBS) were purchased from Corning (Corning, NY). Iba1 primary antibody was purchased from Wako Pure Chemical Corporation (Tokyo, Japan). Goat anti‐rabbit Alexafluor 488 secondary antibody was purchased from Invitrogen (Carlsbad, CA). NucBlue cell stain (DAPI) was purchased from Cell Signaling (Danvers, MA).

### 4.2 Preparation of fluorescently labeled dendrimers

The syntheses of Cy3‐labeled Generation 4 (G4‐Cy3) and Cy5‐labeled Generation 6 dendrimers (G6‐Cy5) were performed as previously described.^25^ Briefly, hydroxyl‐terminated dendrimers were surface‐modified into amine‐terminated bifunctional dendrimers. PyBOP, 6‐Fmoc‐GABA‐OH, and DIEA were combined in anhydrous DMF under nitrogen gas environment for 15 min. Hydroxyl‐terminated G4 and G6 dendrimers were then dissolved in anhydrous DMF and added to the reaction mixture. The reaction ran for 48 hr at room temperature, followed by DMF dialysis for 24 hr. The product was then mixed with piperidine in an ice bath for 15 min. The reagents were removed under reduced pressure until dry. The residue was then dialyzed against DMF for 24 hr and then water for 2 hr. The resulting aqueous solution was lyophilized to yield the dry bifunctional dendrimer. To label the bifunctional dendrimers with Cy3 or Cy5, dendrimers were dissolved in anhydrous DMF and combined with triethylamine and Cy3‐ or Cy5‐mono‐NHS ester. The reaction was run overnight and concentration before extensive purification through DMF and water dialyses. The resulting aqueous solution was lyophilized to yield dry Cy3‐labeled G4 and Cy5‐labeled G6 dendrimers. The final conjugates were characterized by ^1^H‐NMR and HPLC.

### 4.3 Tumor inoculations

All animals were housed at the Johns Hopkins University animal facilities and were given free access to food and water. All animal experiments were approved by the Johns Hopkins University Institutional Animal Care and Use Committee (JHU IACUC).

For the rat model of gliosarcoma, Fischer 344 rats weighing 125–175 g each were implanted intracranially with 9L gliosarcoma as previously described.^22^ Briefly, 9L gliosarcoma tumors were maintained in the flanks of F344 rats, then surgically excised and sectioned into 1 mm^3^ pieces for intracranial implantation. Rats were anesthetized and a midline scalp incision was made. A burr hole 3 mm lateral to the sagittal suture and 5 mm posterior to the coronal suture was made. The dura was incised and, using a surgical microscope and gentle suction, a small cortical area was resected. One tumor section was then placed in the resection cavity, and the skin was closed using surgical staples.

For the mouse model of glioblastoma, male and female C57BL/6 mice 6–8 weeks of age were intracranially implanted with GL261 murine glioblastoma cells. GL261 cells were maintained in RPMI media containing 10% heat inactivated fetal bovine serum, 1% pen/strep antibiotic, and 1% l‐glutamine. Cells were collected via trypsinization and brought to a concentration of 100,000 cells per 2 μl. Mice were anesthetized using a ketamine/xylazine cocktail. A midline scalp incision was made, and a burr hole was drilled 1 mm posterior to the bregma and 2 mm lateral to the midline. A 2 μl Hamilton syringe was lowered to a depth of 2.5 mm into the burr hole to inject 2 μl of cell solution over 10 min. The syringe was then slowly withdrawn, and the incision was sutured together.

### 4.4 Dendrimer administration and tissue processing for biodistribution studies

In the 9L model, 300 μl of 27.5 mg/kg dendrimer doses were systemically administered to rats on day 10 postimplantation. At specified time points (15 min, 8, 24, 48 hr), rats were euthanized, and their major organs were collected and snap frozen in liquid nitrogen. For brain tissues the tumor, peritumor region, and contralateral hemisphere were dissected out for collection. Blood was collected via cardiac puncture and spun down to obtain plasma. For each group, 3–5 animals were used. Dendrimer content in tissue samples were quantified through fluorescence‐based quantification methods as previously described.^22^ Briefly, tissues were dissected into 100 mg sections and homogenized in 1 ml of methanol, followed by sonication for 15 min. Samples were then spun down and supernatants containing extracted dendrimers were collected for quantification. All liquid samples were filtered through a 0.2 μm filter and diluted 10× with PBS.

In the GL261 model, 200 μl of 55 mg/kg dendrimer doses were systemically administered via tail vein to mice on Day 14 postinoculation. At specified time points (1, 4, 24, and 48 hr), organs were collected and snapfrozen in liquid nitrogen. For brains, tumors and tissues from contralateral hemispheres were dissected out. Tissues were dissected into sections (100 mg for livers and kidneys, 50 mg for heart and lungs, 20 mg for spleens) and homogenized in methanol solutions at 100 μl methanol per 10 mg tissue, followed by sonication for 15 min. Samples were then spun down and supernatants collected for analysis. Plasma samples were filtered through a 0.2 μm filter and diluted 5‐fold in PBS.

### 4.5 Fluorescence quantification of dendrimers in tissues

To quantify the G4 or G6 dendrimers in the tissue‐extracted solutions, samples were read on a Shimadzu RF‐5301 Spectro fluorophotometer (Kyoto, Japan). Samples from control tissues were used to correct for background tissue autofluorescence. The wavelengths used for the Cy3‐labeled dendrimers were excitation 554 nm and emission 568 nm. The wavelengths used for the Cy5‐labeled dendrimers were excitation 645 nm and emission 662 nm. Calibration curves to convert measured fluorescence intensities into concentrations were created for each dendrimer. All calibration curves exhibited high linearity with *R* ^2^ > .98.

### 4.6 Confocal imaging

To image dendrimers in the tumor‐bearing brains, animals were euthanized and perfused with saline to wash out residual dendrimers in plasma from the organs. Brains were collected and preserved in 4% formalin solutions overnight, followed by a daily sucrose gradient (10, 20, 30% sucrose in PBS). Brains were then dried and frozen for cryosectioning. Brains were sectioned axially into 30 μm slices using a Leica CM 1905 cryostat (Wetzlar, Germany). The slices were stained with DAPI to visualize cell nuclei and Iba1 to visualize TAMs. Briefly, slices were blocked with 1× TBS + 0.1% Triton X + 1% BSA + 5% NGS for 4 hr, followed by incubation with primary antibody (Iba1 1:200) overnight at 4°C. Then slices were washed and incubated with secondary antibody (goat anti‐rabbit 488 1:200) for 2 hr at room temperature. Finally, slices were incubated with DAPI nuclear stain for 15 min, then mounted and sealed with coverslips.

Dendrimer brain distribution images were obtained using a Zeiss LSM710 confocal microscope (Hertfordshire, UK). Settings were optimized to avoid background fluorescence using untreated animal tissues. Calibration curves for G4 and G6 (Figure S3) showed that for the same concentration, G4 exhibited ~2‐fold greater fluorescence intensity than G6. This difference was offset by adjusting the parameters such as magnification, laser intensity, gain, and offsets in imaging Cy3 and Cy5 channels within each model. Zenlite 2011 software was used to process the obtained images, and any adjustments to brightness and contrast were kept constant across all compared images.

For semi‐quantitative fluorescence analyses of intracellular versus extracellular dendrimer levels in confocal images, ImageJ software was utilized. Intracellular dendrimer signal was determined by selecting TAMs as regions of interest (ROIs) to measure the integrated density parameter. Extracellular signal was determined by setting the entire image as an ROI to quantify the total fluorescence signal and subtracting the intracellular signal. The amount of extracellular signal as a percentage of total dendrimer signal was calculated via the following formula: (total signal – intracellular signal)/total signal. Total tumor signal was determined by selecting the whole tumor in low‐magnification tile scans as an ROI to measure signal intensity.

## 5 CONCLUSIONS

In this study, we explored the effect of increasing dendrimer generation from G4 to G6 on their intrinsic tumor and TAM targeting properties in the 9L rat model of gliosarcoma and the GL261 mouse model of glioblastoma. In these two orthotopic models, both dendrimers achieved high tumor specificity, intrinsic TAMs targeting, and thorough tumor penetration. The larger G6 dendrimers showed significantly enhanced accumulation within the brain tumor (~10‐fold greater at 24 hr) and greater tumor specificity (~2–3‐fold greater) compared to G4 dendrimers. These results demonstrated G6 dendrimers as potentially improved delivery vehicles compared to G4 dendrimers for targeted delivery of immunotherapies into brain tumors, and specifically to TAMs from systemic administration, increasing therapeutic efficacy while reducing side effects. Future work will focus on evaluating how this improved tumor accumulation will translate into superior effects when delivering a therapeutic payload.

## 6 CONFLICT OF INTEREST

The authors have awarded and pending patents relating to TAMs targeting ability of high surface hydroxyl dendrimers. Rangaramanujam M. Kannan and Sujatha Kannan are co‐founders and have financial interests in Ashvattha Therapeutics Inc., Orpheris Inc., and RiniSight, three startups undertaking clinical translation of the dendrimer drug delivery platform.

## Supporting information


**Figure S1** (a) Structure of Generation 4 hydroxyl‐terminated poly(amidoamine) dendrimers. (b) Schematic representation and sizes of Generation 4 (G4) and Generation 6 (G6) dendrimers.

**Figure S2** Generation 6 dendrimers remain in the tumor site but are exported to the extracellular space at 48 hr postinjection. (a) Total fluorescence signal within the tumor remains similar between 24 and 48 hr postinjection for both G4 and G6 dendrimers. (b) G4 and G6 dendrimers exhibit signal within the tumor at 48 hr after injection, with G6 dendrimers exhibiting greater signal throughout the tumor. Green = tumor‐associated macrophage (TAMs), Red = Cy5‐labeled dendrimers, Blue = DAPI nuclear stain. (c) While total tumor signal remains constant from 24 to 48 hr, the location of the dendrimer signal changes for G6 dendrimers over time. The percentage of total tumor signal that is extracellular for G6 dendrimers increases from 17 to 52%. (d) G6 dendrimers exhibit robust extracellular signal (white arrows) at 48 hr postinjection. Green = TAMs, Red = Cy5‐labeled dendrimers.

**Figure S3** The representative calibration curve of G6‐Cy5 (left) and G4‐Cy3 (right). This calibration curve demonstrates G4‐Cy5 is two folds brighter than G6‐Cy5. The calibration curve is generated using a Shimadzu RF‐5301 spectrofluorophotometer. The wavelengths used for the Cy3‐labeled dendrimers are excitation 554 nm and emission 568 nm. The wavelengths used for the Cy5‐labeled dendrimers are excitation 645 nm and emission 662 nm. The calibration curve is generated in methanol under excitation slit width 5 and emission slit width 5.

## References

[1] Wen PY , Kesari S . Malignant gliomas in adults. N Engl J Med. 2008;359(5):492‐507. 18669428 10.1056/NEJMra0708126

[2] Omuro A , DeAngelis LM . Glioblastoma and other malignant gliomas: a clinical review. Jama. 2013;310(17):1842‐1850. 24193082 10.1001/jama.2013.280319

[3] Dolecek TA , Propp JM , Stroup NE , Kruchko C . CBTRUS statistical report: primary brain and central nervous system tumors diagnosed in the United States in 2005‐2009. Neuro Oncol. 2012;14(Suppl 5):v1‐v49. 23095881 10.1093/neuonc/nos218 PMC3480240

[4] McGirt MJ , Than KD , Weingart JD , et al. Gliadel (BCNU) wafer plus concomitant temozolomide therapy after primary resection of glioblastoma multiforme. J Neurosurg. 2009;110(3):583‐588. 19046047 10.3171/2008.5.17557 PMC4856017

[5] Chowdhary SA , Ryken T , Newton HB . Survival outcomes and safety of carmustine wafers in the treatment of high‐grade gliomas: a meta‐analysis. J Neurooncol. 2015;122(2):367‐382. 25630625 10.1007/s11060-015-1724-2 PMC4368843

[6] Mehta M , Wen P , Nishikawa R , Reardon D , Peters K . Critical review of the addition of tumor treating fields (TTFields) to the existing standard of care for newly diagnosed glioblastoma patients. Crit Rev Oncol Hematol. 2017;111:60‐65. 28259296 10.1016/j.critrevonc.2017.01.005

[7] Sarin H , Kanevsky AS , Wu H , et al. Physiologic upper limit of pore size in the blood‐tumor barrier of malignant solid tumors. J Transl Med. 2009;7(1):51. 19549317 10.1186/1479-5876-7-51 PMC2706803

[8] Jain RK , Stylianopoulos T . Delivering nanomedicine to solid tumors. Nat Rev Clin Oncol. 2010;7(11):653‐664. 20838415 10.1038/nrclinonc.2010.139 PMC3065247

[9] Sun Q , Ojha T , Kiessling F , Lammers T , Shi Y . Enhancing tumor penetration of nanomedicines. Biomacromolecules. 2017;18(5):1449‐1459. 28328191 10.1021/acs.biomac.7b00068 PMC5424079

[10] Sarin H , Kanevsky AS , Wu H , et al. Effective transvascular delivery of nanoparticles across the blood‐brain tumor barrier into malignant glioma cells. J Transl Med. 2008;6:80. 19094226 10.1186/1479-5876-6-80 PMC2639552

[11] Longmire M , Choyke PL , Kobayashi H . Clearance properties of nano‐sized particles and molecules as imaging agents: considerations and caveats. Nanomedicine (Lond). 2008;3(5):703‐717. 18817471 10.2217/17435889.3.5.703 PMC3407669

[12] Nichols JW , Bae YH . Odyssey of a cancer nanoparticle: from injection site to site of action. Nano Today. 2012;7(6):606‐618. 23243460 10.1016/j.nantod.2012.10.010 PMC3519442

[13] Pathria P , Louis TL , Varner JA . Targeting tumor‐associated macrophages in cancer. Trends Immunol. 2019;40(4):310‐327. 30890304 10.1016/j.it.2019.02.003

[14] Lin Y , Xu J , Lan H . Tumor‐associated macrophages in tumor metastasis: biological roles and clinical therapeutic applications. J Hematol Oncol. 2019;12(1):76. 31300030 10.1186/s13045-019-0760-3 PMC6626377

[15] Jeong H , Kim S , Hong B‐J , et al. Tumor‐associated macrophages enhance tumor hypoxia and aerobic glycolysis. Cancer res. 2019;79(4):795‐806. 30610087 10.1158/0008-5472.CAN-18-2545

[16] Fu X‐T , Song K , Zhou J , et al. Tumor‐associated macrophages modulate resistance to oxaliplatin via inducing autophagy in hepatocellular carcinoma. Cancer Cell Int. 2019;19(1):71. 30962765 10.1186/s12935-019-0771-8 PMC6434873

[17] Zhang F , Parayath NN , Ene CI , et al. Genetic programming of macrophages to perform anti‐tumor functions using targeted mRNA nanocarriers. Nat Commun. 2019;10(1):3974. 31481662 10.1038/s41467-019-11911-5 PMC6722139

[18] Mantovani A , Marchesi F , Malesci A , Laghi L , Allavena P . Tumour‐associated macrophages as treatment targets in oncology. Nat Rev Clin Oncol. 2017;14(7):399‐416. 28117416 10.1038/nrclinonc.2016.217 PMC5480600

[19] Sauter KA , Pridans C , Sehgal A , et al. Pleiotropic effects of extended blockade of CSF1R signaling in adult mice. J Leukoc Biol. 2014;96(2):265‐274. 24652541 10.1189/jlb.2A0114-006R PMC4378363

[20] Tap WD , Gelderblom H , Stacchiotti S , et al. Final results of ENLIVEN: a global, double‐blind, randomized, placebo‐controlled, phase 3 study of pexidartinib in advanced tenosynovial giant cell tumor (TGCT). J Clin Oncol. 2018;36(15_suppl):11502‐11502.

[21] Radi ZA , Koza‐Taylor PH , Bell RR , et al. Increased serum enzyme levels associated with Kupffer cell reduction with no signs of hepatic or skeletal muscle injury. Am J Pathol. 2011;179(1):240‐247. 21703406 10.1016/j.ajpath.2011.03.029 PMC3123844

[22] Zhang F , Mastorakos P , Mishra MK , et al. Uniform brain tumor distribution and tumor associated macrophage targeting of systemically administered dendrimers. Biomaterials. 2015;52:507‐516. 25818456 10.1016/j.biomaterials.2015.02.053 PMC4710089

[23] Liu X , Liu C , Catapano CV , Peng L , Zhou J , Rocchi P . Structurally flexible triethanolamine‐core poly(amidoamine) dendrimers as effective nanovectors to deliver RNAi‐based therapeutics. Biotechnol Adv. 2014;32(4):844‐852. 23938269 10.1016/j.biotechadv.2013.08.001

[24] Gonawala S , Ali MM . Application of dendrimer‐based nanoparticles in glioma imaging. J Nanomed Nanotechnol. 2017;8:3. 10.4172/2157-7439.1000444 PMC5573247 28856067

[25] Lesniak WG , Mishra MK , Jyoti A , et al. Biodistribution of fluorescently labeled PAMAM dendrimers in neonatal rabbits: effect of neuroinflammation. Mol Pharm. 2013;10(12):4560‐4571. 24116950 10.1021/mp400371r PMC3977004

[26] Mishra MK , Beaty CA , Lesniak WG , et al. Dendrimer brain uptake and targeted therapy for brain injury in a large animal model of hypothermic circulatory arrest. ACS Nano. 2014;8(3):2134‐2147. 24499315 10.1021/nn404872e PMC4004292

[27] Nance E , Kambhampati SP , Smith ES , et al. Dendrimer‐mediated delivery of N‐acetyl cysteine to microglia in a mouse model of Rett syndrome. J Neuroinflammation. 2017;14(1):252. 29258545 10.1186/s12974-017-1004-5 PMC5735803

[28] Sharma A , Porterfield JE , Smith E , Sharma R , Kannan S , Kannan RM . Effect of mannose targeting of hydroxyl PAMAM dendrimers on cellular and organ biodistribution in a neonatal brain injury model. J Control Rel. 2018;283:175‐189. 10.1016/j.jconrel.2018.06.003 PMC6091673 29883694

[29] Nino DF , Zhou Q , Yamaguchi Y , et al. Cognitive impairments induced by necrotizing enterocolitis can be prevented by inhibiting microglial activation in mouse brain. Sci Transl Med. 2018;10(471):eaan0237. 30541786 10.1126/scitranslmed.aan0237 PMC8170511

[30] Dobrovolskaia MA . Dendrimers effects on the immune system: insights into toxicity and therapeutic utility. Curr Pharm des. 2017;23(21):3134‐3141. 28294045 10.2174/1381612823666170309151958

[31] Sharma R , Sharma A , Kambhampati SP , et al. Scalable synthesis and validation of PAMAM dendrimer‐N‐acetyl cysteine conjugate for potential translation. Bioeng Transl Med. 2018;3(2):87‐101. 30065965 10.1002/btm2.10094 PMC6063872

[32] Barth RF , Kaur B . Rat brain tumor models in experimental neuro‐oncology: the C6, 9L, T9, RG2, F98, BT4C, RT‐2 and CNS‐1 gliomas. J Neurooncol. 2009;94(3):299‐312. 19381449 10.1007/s11060-009-9875-7 PMC2730996

[33] Oh T , Fakurnejad S , Sayegh ET , et al. Immunocompetent murine models for the study of glioblastoma immunotherapy. J Transl Med. 2014;12:107. 24779345 10.1186/1479-5876-12-107 PMC4012243

[34] Zhu Y , Liu C , Pang Z . Dendrimer‐based drug delivery systems for brain targeting. Biomolecules. 2019;9(12):790. 10.3390/biom9120790 PMC6995517 31783573

[35] Zhang F , Trent Magruder J , Lin YA , et al. Generation‐6 hydroxyl PAMAM dendrimers improve CNS penetration from intravenous administration in a large animal brain injury model. J Control Rel. 2017;249:173‐182. 10.1016/j.jconrel.2017.01.032 PMC5323327 28137632

[36] Goodman TT , Olive PL , Pun SH . Increased nanoparticle penetration in collagenase‐treated multicellular spheroids. Int J Nanomedicine. 2007;2(2):265‐274. 17722554 PMC2673974

[37] Tang L , Yang X , Yin Q , et al. Investigating the optimal size of anticancer nanomedicine. Proc Natl Acad Sci U S A. 2014;111(43):15344‐15349. 25316794 10.1073/pnas.1411499111 PMC4217425

[38] Liu D , Mori A , Huang L . Role of liposome size and RES blockade in controlling biodistribution and tumor uptake of GM1‐containing liposomes. Biochim Biophys Acta. 1992;1104(1):95‐101. 1550858 10.1016/0005-2736(92)90136-a

[39] Hobbs SK , Monsky WL , Yuan F , et al. Regulation of transport pathways in tumor vessels: role of tumor type and microenvironment. Proc Natl Acad Sci U S A. 1998;95(8):4607‐4612. 9539785 10.1073/pnas.95.8.4607 PMC22537

[40] Yu M , Zheng J . Clearance pathways and tumor targeting of imaging nanoparticles. ACS Nano. 2015;9(7):6655‐6674. 26149184 10.1021/acsnano.5b01320 PMC4955575

[41] Choi HS , Liu W , Misra P , et al. Renal clearance of quantum dots. Nat Biotechnol. 2007;25(10):1165‐1170. 17891134 10.1038/nbt1340 PMC2702539

[42] Moeller MJ , Tenten V . Renal albumin filtration: alternative models to the standard physical barriers. Nat Rev Nephrol. 2013;9(5):266‐277. 23528417 10.1038/nrneph.2013.58

[43] Hausmann R , Kuppe C , Egger H , et al. Electrical forces determine glomerular permeability. J Am Soc Nephrol. 2010;21(12):2053‐2058. 20947631 10.1681/ASN.2010030303 PMC3014018

[44] Smithies O . Why the kidney glomerulus does not clog: a gel permeation/diffusion hypothesis of renal function. Proc Natl Acad Sci U S A. 2003;100(7):4108‐4113. 12655073 10.1073/pnas.0730776100 PMC153056

[45] Hoshyar N , Gray S , Han H , Bao G . The effect of nanoparticle size on in vivo pharmacokinetics and cellular interaction. Nanomedicine (Lond). 2016;11(6):673‐692. 27003448 10.2217/nnm.16.5 PMC5561790

[46] Jiang Y , Lv L , Shi H , et al. PEGylated Polyamidoamine dendrimer conjugated with tumor homing peptide as a potential targeted delivery system for glioma. Colloids Surf B Biointerfaces. 2016;147:242‐249. 27518456 10.1016/j.colsurfb.2016.08.002

[47] Karki K , Ewing JR , Ali MM . Targeting glioma with a dual mode optical and paramagnetic Nanoprobe across the blood‐brain tumor barrier. J Nanomed Nanotechnol. 2016;7(4):395. 27695645 10.4172/2157-7439.1000395 PMC5042151

[48] Zhao J , Zhang B , Shen S , et al. CREKA peptide‐conjugated dendrimer nanoparticles for glioblastoma multiforme delivery. J Colloid Interface Sci. 2015;450:396‐403. 25863222 10.1016/j.jcis.2015.03.019

[49] Mehta D , Leong N , McLeod VM , et al. Reducing dendrimer generation and PEG chain length increases drug release and promotes anticancer activity of PEGylated Polylysine dendrimers conjugated with doxorubicin via a Cathepsin‐cleavable peptide linker. Mol Pharm. 2018;15(10):4568‐4576. 30107748 10.1021/acs.molpharmaceut.8b00581

[50] Garrigue P , Tang J , Ding L , et al. Self‐assembling supramolecular dendrimer nanosystem for PET imaging of tumors. Proc Natl Acad Sci U S A. 2018;115(45):11454‐11459. 30348798 10.1073/pnas.1812938115 PMC6233080

[51] Hu G , Zhang H , Zhang L , Ruan S , He Q , Gao H . Integrin‐mediated active tumor targeting and tumor microenvironment response dendrimer‐gelatin nanoparticles for drug delivery and tumor treatment. Int J Pharm. 2015;496(2):1057‐1068. 26598487 10.1016/j.ijpharm.2015.11.025

[52] Serwer LP , Noble CO , Michaud K , et al. Investigation of intravenous delivery of nanoliposomal topotecan for activity against orthotopic glioblastoma xenografts. Neuro Oncol. 2011;13(12):1288‐1295. 21954443 10.1093/neuonc/nor139 PMC3223095

[53] Peng C , Gao X , Xu J , et al. Targeting orthotopic gliomas with renal‐clearable luminescent gold nanoparticles. Nano Res. 2017;10(4):1366‐1376. 29034063 10.1007/s12274-017-1472-z PMC5639726

[54] Coluccia D , Figueiredo CA , Wu MY , et al. Enhancing glioblastoma treatment using cisplatin‐gold‐nanoparticle conjugates and targeted delivery with magnetic resonance‐guided focused ultrasound. Nanomedicine: Nanotechnology, Biology and Medicine. 2018;14(4):1137‐1148. 10.1016/j.nano.2018.01.021 29471172

[55] Ganipineni LP , Ucakar B , Joudiou N , et al. Magnetic targeting of paclitaxel‐loaded poly(lactic‐co‐glycolic acid)‐based nanoparticles for the treatment of glioblastoma. Int J Nanomedicine. 2018;13:4509‐4521. 30127603 10.2147/IJN.S165184 PMC6092128

[56] Tian XH , Lin XN , Wei F , et al. Enhanced brain targeting of temozolomide in polysorbate‐80 coated polybutylcyanoacrylate nanoparticles. Int J Nanomedicine. 2011;6:445‐452. 21445277 10.2147/IJN.S16570 PMC3061435

[57] Joshi S , Cooke JR , Chan DK , et al. Liposome size and charge optimization for intraarterial delivery to gliomas. Drug Deliv Transl res. 2016;6(3):225‐233. 27091339 10.1007/s13346-016-0294-y PMC5508862

[58] Kreuter J . Drug delivery to the central nervous system by polymeric nanoparticles: what do we know? Adv Drug Deliv Rev. 2014;71:2‐14. 23981489 10.1016/j.addr.2013.08.008

[59] Zou Y , Liu Y , Yang Z , et al. Effective and targeted human Orthotopic glioblastoma xenograft therapy via a multifunctional biomimetic nanomedicine. Adv Mater (Deerfield Beach, Fla.). 2018;30(51):e1803717. 10.1002/adma.201803717 30328157

[60] Prabhu S , Goda JS , Mutalik S , et al. A polymeric temozolomide nanocomposite against orthotopic glioblastoma xenograft: tumor‐specific homing directed by nestin. Nanoscale. 2017;9(30):10919‐10932. 28731079 10.1039/c7nr00305f

[61] Galanis E , Buckner JC , Dinapoli RP , et al. Clinical outcome of gliosarcoma compared with glioblastoma multiforme: north central cancer treatment group results. J Neurosurg. 1998;89(3):425‐430. 9724117 10.3171/jns.1998.89.3.0425

[62] Lee EQ , Kuhn J , Lamborn KR , et al. Phase I/II study of sorafenib in combination with temsirolimus for recurrent glioblastoma or gliosarcoma: north American brain tumor consortium study 05‐02. Neuro Oncol. 2012;14(12):1511‐1518. 23099651 10.1093/neuonc/nos264 PMC3499017

[63] Prados MD , Chang SM , Butowski N , et al. Phase II study of erlotinib plus temozolomide during and after radiation therapy in patients with newly diagnosed glioblastoma multiforme or gliosarcoma. J Clin Oncol. 2009;27(4):579‐584. 19075262 10.1200/JCO.2008.18.9639 PMC2645859

[64] Zhang G , Huang S , Zhang J , Wu Z , Lin S , Wang Y . Clinical outcome of gliosarcoma compared with glioblastoma multiforme: a clinical study in Chinese patients. J Neurooncol. 2016;127(2):355‐362. 26725096 10.1007/s11060-015-2046-0

[65] Han SJ , Yang I , Tihan T , Prados MD , Parsa AT . Primary gliosarcoma: key clinical and pathologic distinctions from glioblastoma with implications as a unique oncologic entity. J Neurooncol. 2010;96(3):313‐320. 19618114 10.1007/s11060-009-9973-6 PMC2808523

[66] Seunggu JH , Isaac Y , Jose JO , et al. Secondary gliosarcoma after diagnosis of glioblastoma: clinical experience with 30 consecutive patients. J Neurosurg. 2010;112(5):990‐996. 19817543 10.3171/2009.9.JNS09931

[67] Nakagomi T , Goto T , Hirotsu Y , et al. New therapeutic targets for pulmonary sarcomatoid carcinomas based on their genomic and phylogenetic profiles. Oncotarget. 2018;9(12):10635‐10649. 29535832 10.18632/oncotarget.24365 PMC5828205

[68] Roszer T . Understanding the mysterious M2 macrophage through activation markers and effector mechanisms. Mediators Inflamm. 2015;2015:16. 10.1155/2015/816460 PMC4452191 26089604

[69] Edin S , Wikberg M , Rutegård J , Oldenborg P‐A , Palmqvist R . Phenotypic skewing of macrophages in vitro by secreted factors from colorectal cancer cells. PloS One. 2013;8:e74982. 24058644 10.1371/journal.pone.0074982 PMC3776729

[70] Zhang B , Shen R , Cheng S , Feng L . Immune microenvironments differ in immune characteristics and outcome of glioblastoma multiforme. Cancer Med. 2019;8(6):2897‐2907. 31038851 10.1002/cam4.2192 PMC6558448

[71] Rabe DC , Rustandy FD , Lee J , Rosner MR . Tumor extracellular vesicles are required for tumor‐associated macrophage programming. *bioRxiv* 2018; 375022.

[72] Wen C , Seeger RC , Fabbri M , Wang L , Wayne AS , Jong AY . Biological roles and potential applications of immune cell‐derived extracellular vesicles. J Extracell Ves. 2017;6(1):1400370. 10.1080/20013078.2017.1400370 PMC5706476 29209467

[73] Buzás EI , Tóth EÁ , Sódar BW , Szabó‐Taylor KÉ . Molecular interactions at the surface of extracellular vesicles. Semin Immunopathol. 2018;40(5):453‐464. 29663027 10.1007/s00281-018-0682-0 PMC6208672

[74] Kroschinsky F , Stölzel F , von Bonin S , et al. New drugs, new toxicities: severe side effects of modern targeted and immunotherapy of cancer and their management. Crit Care. 2017;21(1):89. 28407743 10.1186/s13054-017-1678-1 PMC5391608

[75] Weber JS , Yang JC , Atkins MB , Disis ML . Toxicities of immunotherapy for the practitioner. J Clin Oncol. 2015;33(18):2092‐2099. 25918278 10.1200/JCO.2014.60.0379 PMC4881375

[76] Jain D , Bajaj A , Athawale R , et al. Surface‐coated PLA nanoparticles loaded with temozolomide for improved brain deposition and potential treatment of gliomas: development, characterization and in vivo studies. Drug Deliv. 2016;23(3):989‐1006. 10.3109/10717544.2014.926574 25026415

[77] Song J , Yang X , Jacobson O , et al. Ultrasmall gold Nanorod vesicles with enhanced tumor accumulation and fast excretion from the body for cancer therapy. Adv Mater. 2015;27(33):4910‐4917. 26198622 10.1002/adma.201502486

