# Supplementary material for: Dendrimer size effects on the selective brain tumor targeting in orthotopic tumor models upon systemic administration
Source: Bioeng Transl Med. 2020 Apr 14;5(2):e10160. doi: 10.1002/btm2.10160 (PMC7237147; doi:10.1002/btm2.10160)
Supplement: Supplementary file 1 — Figure S1 (a) Structure of Generation 4 hydroxyl‐terminated poly(amidoamine) dendrimers. (b) Schematic representation and sizes of Generation 4 (G4) and Generation 6 (G6) dendrimers. [file BTM2-5-e10160-s001.pptx]

## Slide 1
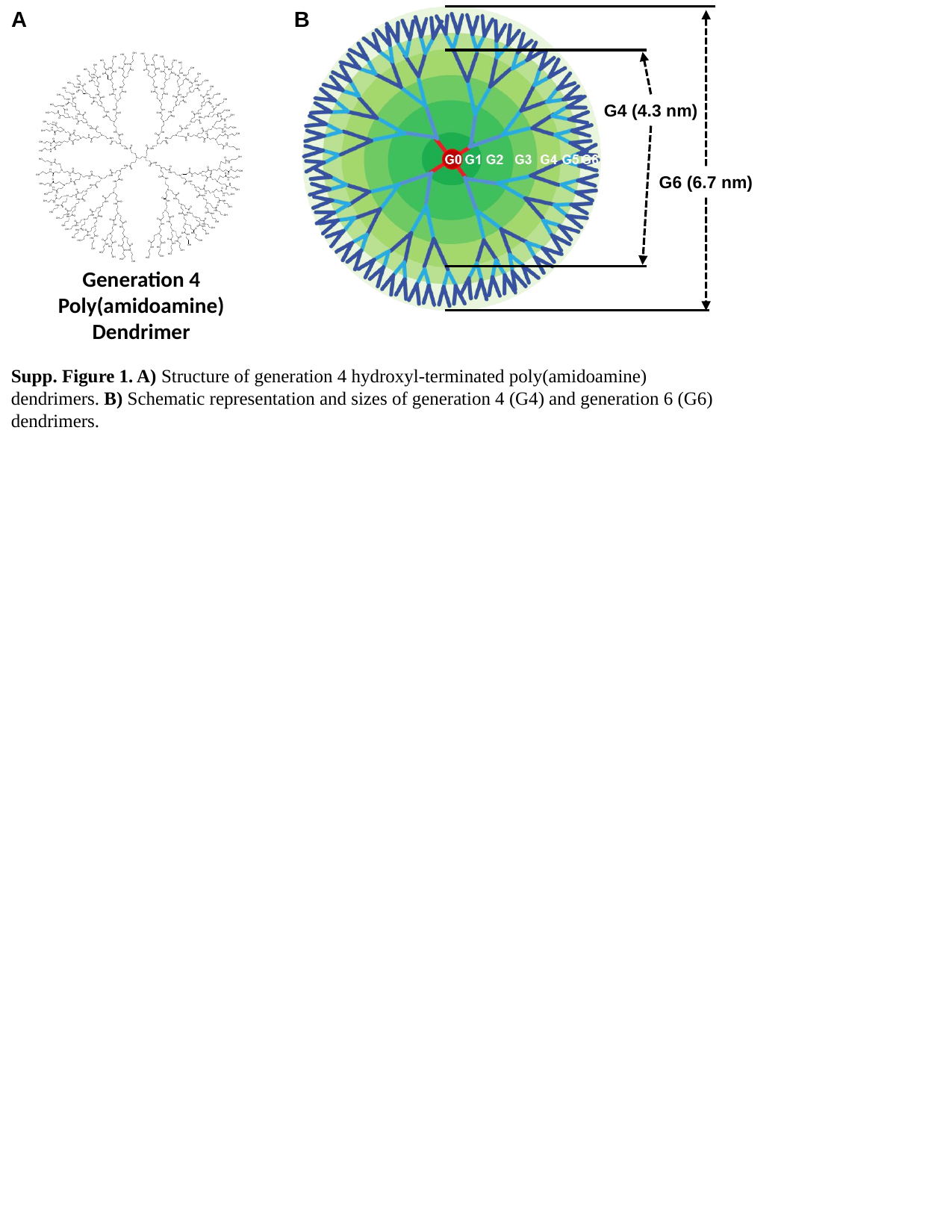

B
A
G4 (4.3 nm)
G6 (6.7 nm)
Generation 4 Poly(amidoamine) Dendrimer
Supp. Figure 1. A) Structure of generation 4 hydroxyl-terminated poly(amidoamine) dendrimers. B) Schematic representation and sizes of generation 4 (G4) and generation 6 (G6) dendrimers.
