# Supplementary material for: Dendrimer size effects on the selective brain tumor targeting in orthotopic tumor models upon systemic administration
Source: Bioeng Transl Med. 2020 Apr 14;5(2):e10160. doi: 10.1002/btm2.10160 (PMC7237147; doi:10.1002/btm2.10160)
Supplement: Supplementary file 2 — Figure S2 Generation 6 dendrimers remain in the tumor site but are exported to the extracellular space at 48 hr postinjection. (a) Total fluorescence signal within the tumor remains similar between 24 and 48 hr postinjection for both G4 and G6 dendrimers. (b) G4 and G6 dendrimers exhibit signal within the tumor at 48 hr after injection, with G6 dendrimers exhibiting greater signal throughout the tumor. Green = tumor‐associated macrophage (TAMs), Red = Cy5‐labeled dendrimers, Blue = DAPI nuclear stain. (c) While total tumor signal remains constant from 24 to 48 hr, the location of the dendrimer signal changes for G6 dendrimers over time. The percentage of total tumor signal that is extracellular for G6 dendrimers increases from 17 to 52%. (d) G6 dendrimers exhibit robust extracellular signal (white arrows) at 48 hr postinjection. Green = TAMs, Red = Cy5‐labeled dendrimers. [file BTM2-5-e10160-s002.pptx]

## Slide 1
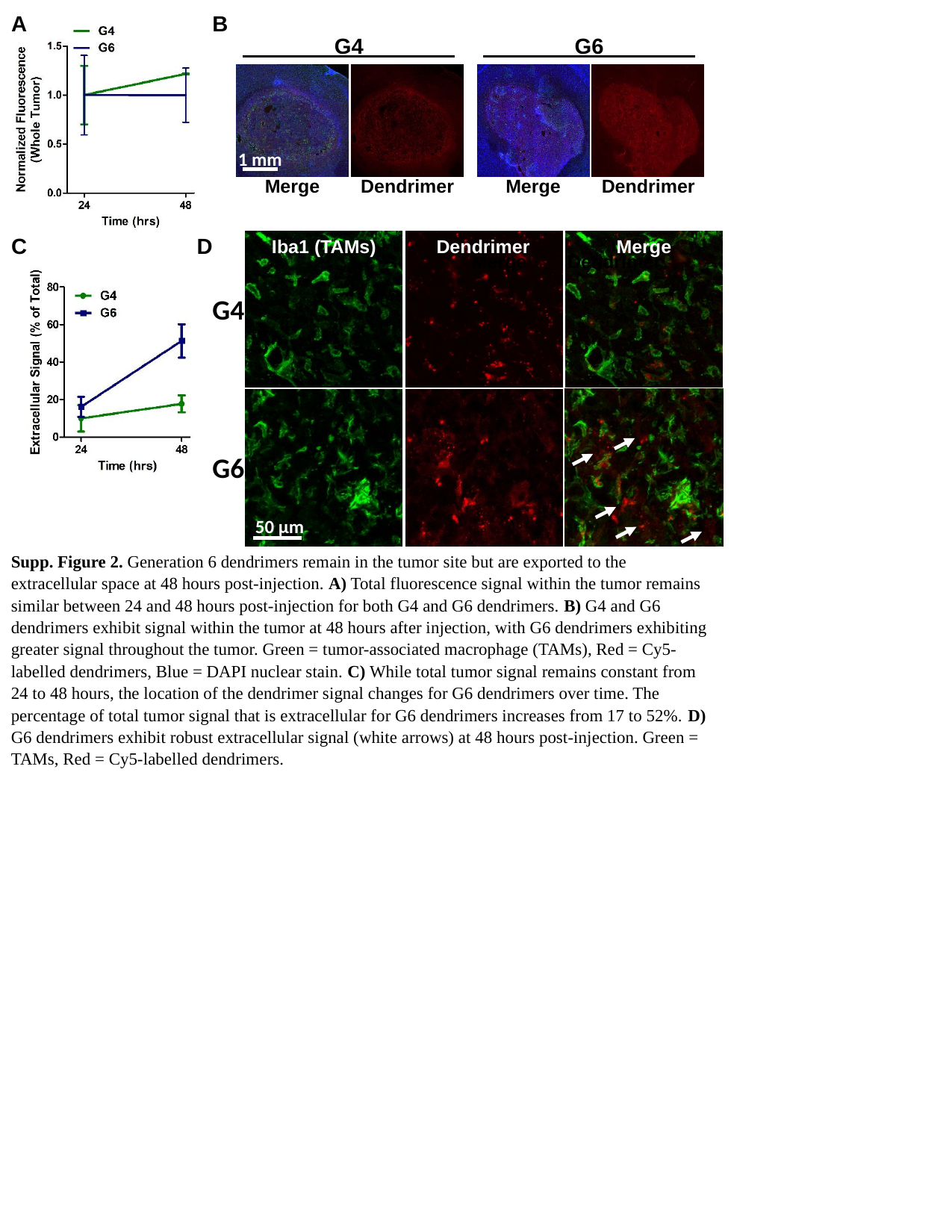

A
B
G4
G6
1 mm
Merge
Dendrimer
Merge
Dendrimer
C
D
Iba1 (TAMs)
Dendrimer
Merge
Merge
Dendrimer
G4
G6
50 µm
Supp. Figure 2. Generation 6 dendrimers remain in the tumor site but are exported to the extracellular space at 48 hours post-injection. A) Total fluorescence signal within the tumor remains similar between 24 and 48 hours post-injection for both G4 and G6 dendrimers. B) G4 and G6 dendrimers exhibit signal within the tumor at 48 hours after injection, with G6 dendrimers exhibiting greater signal throughout the tumor. Green = tumor-associated macrophage (TAMs), Red = Cy5-labelled dendrimers, Blue = DAPI nuclear stain. C) While total tumor signal remains constant from 24 to 48 hours, the location of the dendrimer signal changes for G6 dendrimers over time. The percentage of total tumor signal that is extracellular for G6 dendrimers increases from 17 to 52%. D) G6 dendrimers exhibit robust extracellular signal (white arrows) at 48 hours post-injection. Green = TAMs, Red = Cy5-labelled dendrimers.
