# Supplementary material for: Dendrimer size effects on the selective brain tumor targeting in orthotopic tumor models upon systemic administration
Source: Bioeng Transl Med. 2020 Apr 14;5(2):e10160. doi: 10.1002/btm2.10160 (PMC7237147; doi:10.1002/btm2.10160)
Supplement: Supplementary file 3 — Figure S3 The representative calibration curve of G6‐Cy5 (left) and G4‐Cy3 (right). This calibration curve demonstrates G4‐Cy5 is two folds brighter than G6‐Cy5. The calibration curve is generated using a Shimadzu RF‐5301 spectrofluorophotometer. The wavelengths used for the Cy3‐labeled dendrimers are excitation 554 nm and emission 568 nm. The wavelengths used for the Cy5‐labeled dendrimers are excitation 645 nm and emission 662 nm. The calibration curve is generated in methanol under excitation slit width 5 and emission slit width 5. [file BTM2-5-e10160-s003.pptx]

## Slide 1
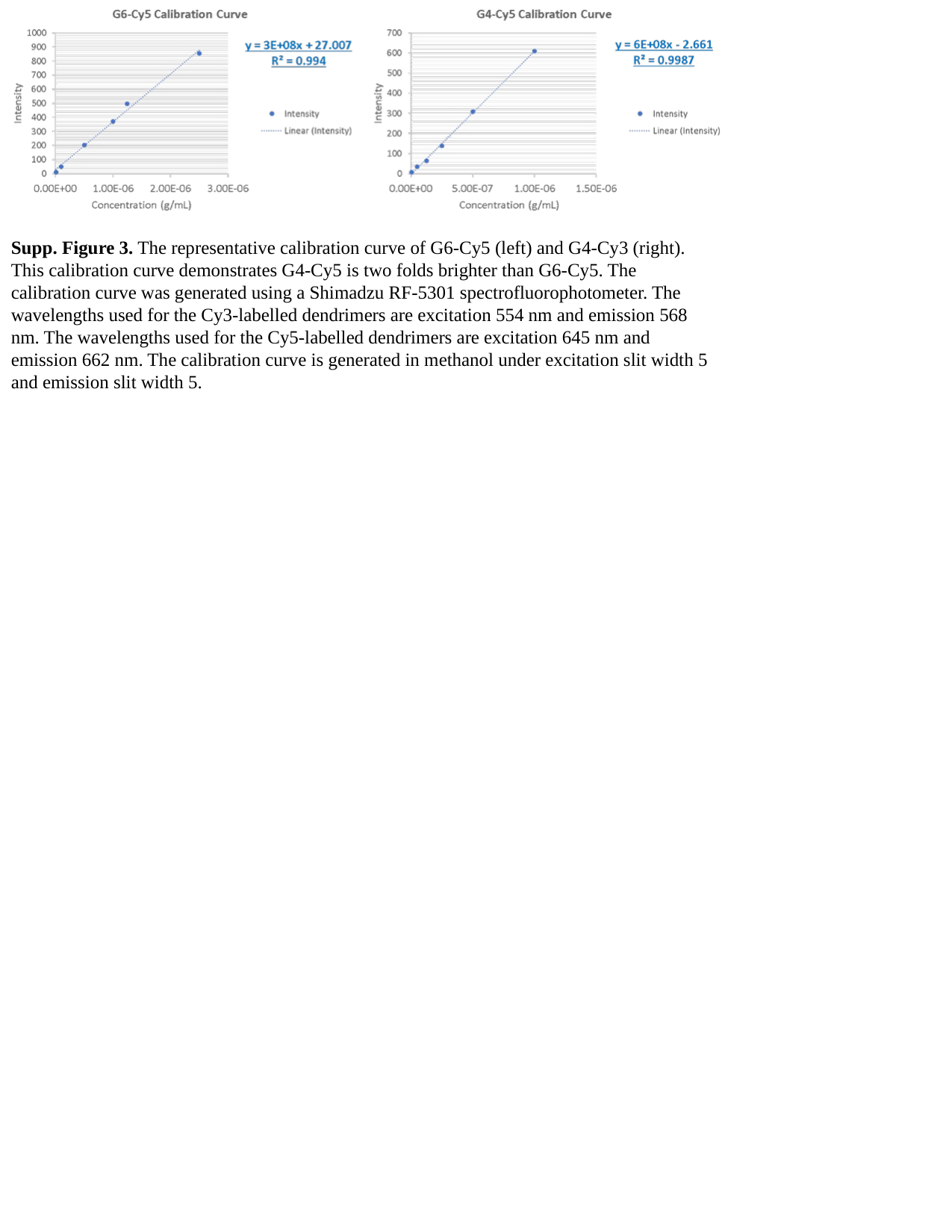

Supp. Figure 3. The representative calibration curve of G6-Cy5 (left) and G4-Cy3 (right). This calibration curve demonstrates G4-Cy5 is two folds brighter than G6-Cy5. The calibration curve was generated using a Shimadzu RF-5301 spectrofluorophotometer. The wavelengths used for the Cy3-labelled dendrimers are excitation 554 nm and emission 568 nm. The wavelengths used for the Cy5-labelled dendrimers are excitation 645 nm and emission 662 nm. The calibration curve is generated in methanol under excitation slit width 5 and emission slit width 5.
